# Supplementary material for: Low-dose VSV-EBOV vaccination provides rapid protection from lethal Ebola virus challenge
Source: bioRxiv. 2026 Feb 15:2026.02.14.705917. Preprint. [Version 1] doi: 10.64898/2026.02.14.705917 (PMC12919010; doi:10.64898/2026.02.14.705917)
Supplement: Supplement 1 [file NIHPP2026.02.14.705917v1-supplement-1.pdf]

bioRxiv preprint doi: https://doi.org/10.64898/2026.02.14.705917; this version posted February 15, 2026. The copyright holder for this preprint (which was not certified by peer review) is the author/funder. This article is a US Government work. It is not subject to copyright under 17 USC 105 and is also made available for use under a CC0 license.

Supplemental information

Figure S1. Study design and VSV RNA circulation after vaccination. (A) Study groups are depicted. The animal in the control group (gray) that died due to anesthetic complications is indicated in black. **(B)** Vaccination (gray arrow) and challenge schedule (open arrow) for the study. Triangles indicate physical exams including blood draw. **(C)** VSV RNA in the blood of vaccinated NHPs before challenge. BL base line. Statistical significance is indicated (* $p< 0.05$).

Figure S2. Serum chemistry after EBOV infection in NHPs. The levels of **(A)** alanine aminotransferase (ALP), **(B)** aspartate aminotransferase (AST), **(C)** alkaline phosphatase (ALP), **(D)** blood urea nitrogen (BUN), and **(E)** creatinine (CRE) were determined from serum samples collected after challenge.

Figure S3. Fc effort function analysis of serum. Levels of **(A)** antibody-dependent complement deposition (ADCD) and **(B)** antibody-dependent natural killer cell activation (ADNKA) were determined at selected time points. Statistical significance is indicated (* $p< 0.05$).

Figure S4. VSV vaccine characterization *in vitro*. Western blot analysis of **(A)** virus stock and **(B)** 1x 10^4 PFU matched titers of VSVs. Lane 1: uninfected Vero E6 cell supernatant; lane 2: VSV wildtype (non-GMP-grade); lane 3: VSV-$EBOV_{Mak}$ (lane 3; non-GMP-grade); lane 4: VSV-$EBOV_{Kik}$ (lane 4; GMP-grade). Mouse monoclonal antibodies specific to EBOV GP and VSV M were used. **(C)** VSV growth kinetics at a MOI=0.001 on Vero E6 cells. Statistical significance is indicated (* $p< 0.05$).

Figure S1

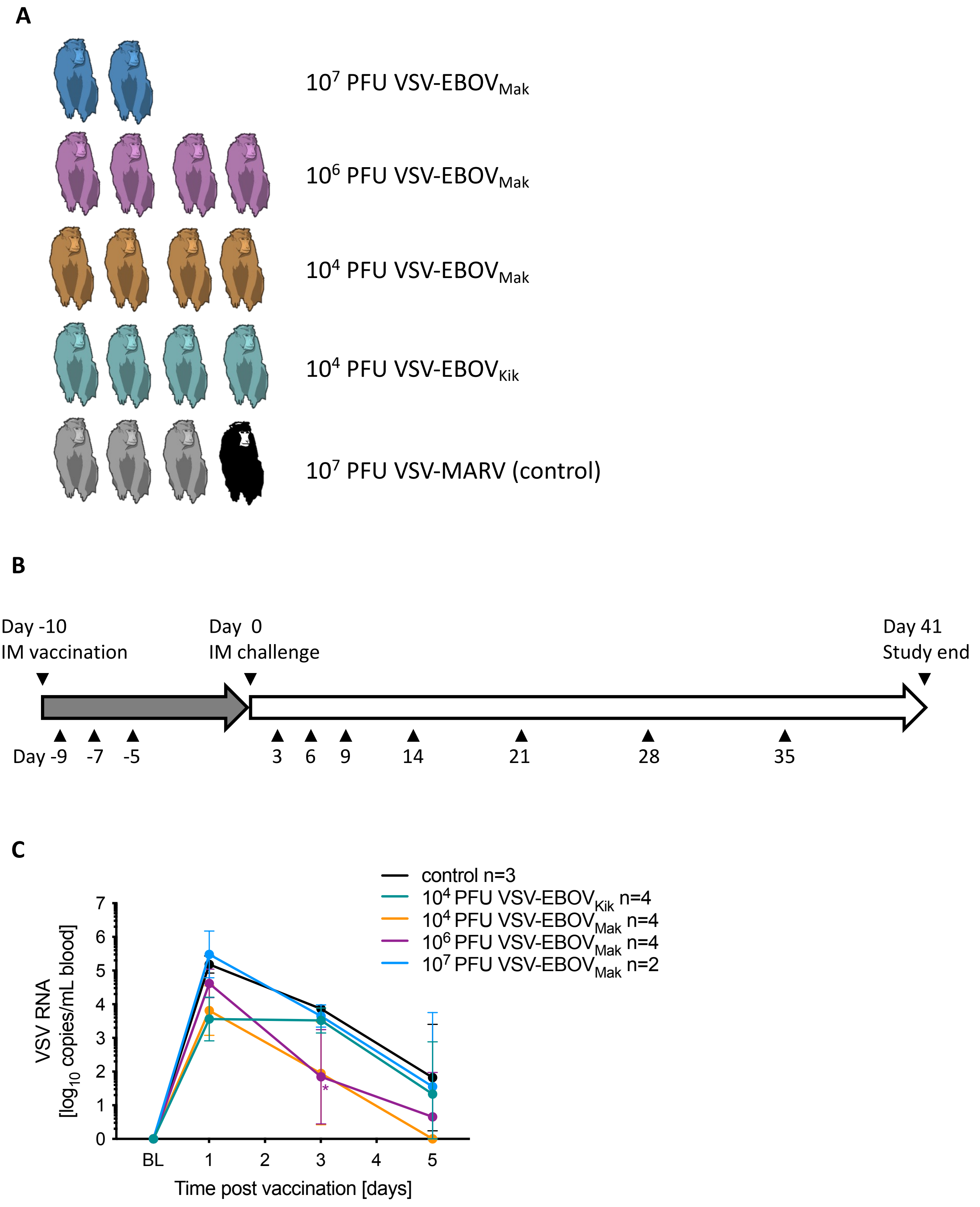

A
10^7 PFU VSV-EBOV_Mak
10^6 PFU VSV-EBOV_Mak
10^4 PFU VSV-EBOV_Mak
10^4 PFU VSV-EBOV_Kik
10^7 PFU VSV-MARV (control)
B
Day -10
IM vaccination
Day 0
IM challenge
Day 41
Study end
Day -9
-7
-5
3
6
9
14
21
28
35
C
control n=3
10^4 PFU VSV-EBOV_Kik n=4
10^4 PFU VSV-EBOV_Mak n=4
10^6 PFU VSV-EBOV_Mak n=4
10^7 PFU VSV-EBOV_Mak n=2
VSV RNA [log10 copies/mL blood]
0
1
2
3
4
5
6
7
BL
1
2
3
4
5
Time post vaccination [days]
*

Figure S2

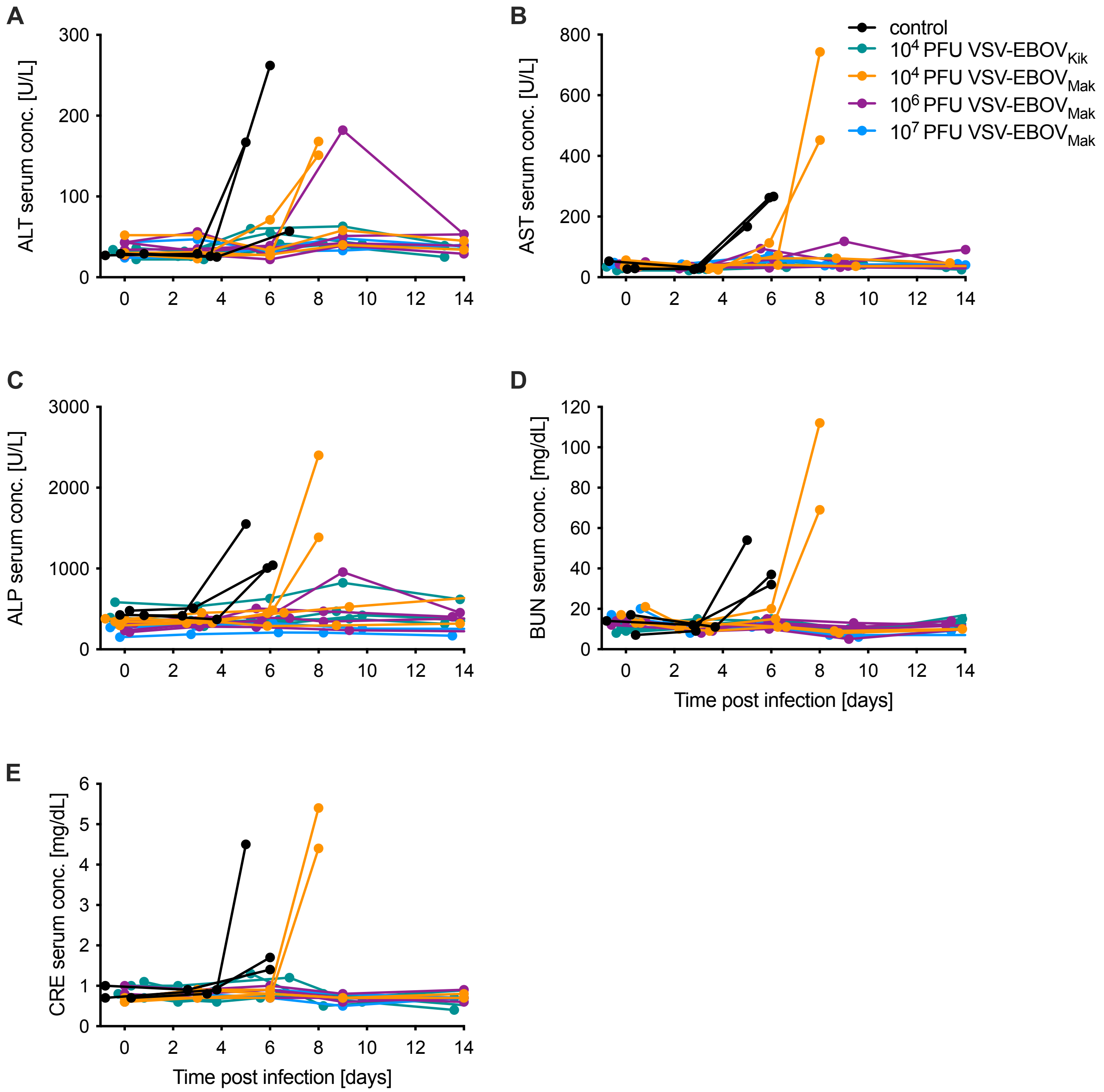

A
B
C
D
E
control
10^4 PFU VSV-EBOV_Kik
10^4 PFU VSV-EBOV_Mak
10^6 PFU VSV-EBOV_Mak
10^7 PFU VSV-EBOV_Mak
ALT serum conc. [U/L]
AST serum conc. [U/L]
ALP serum conc. [U/L]
BUN serum conc. [mg/dL]
CRE serum conc. [mg/dL]
Time post infection [days]

Figure S3

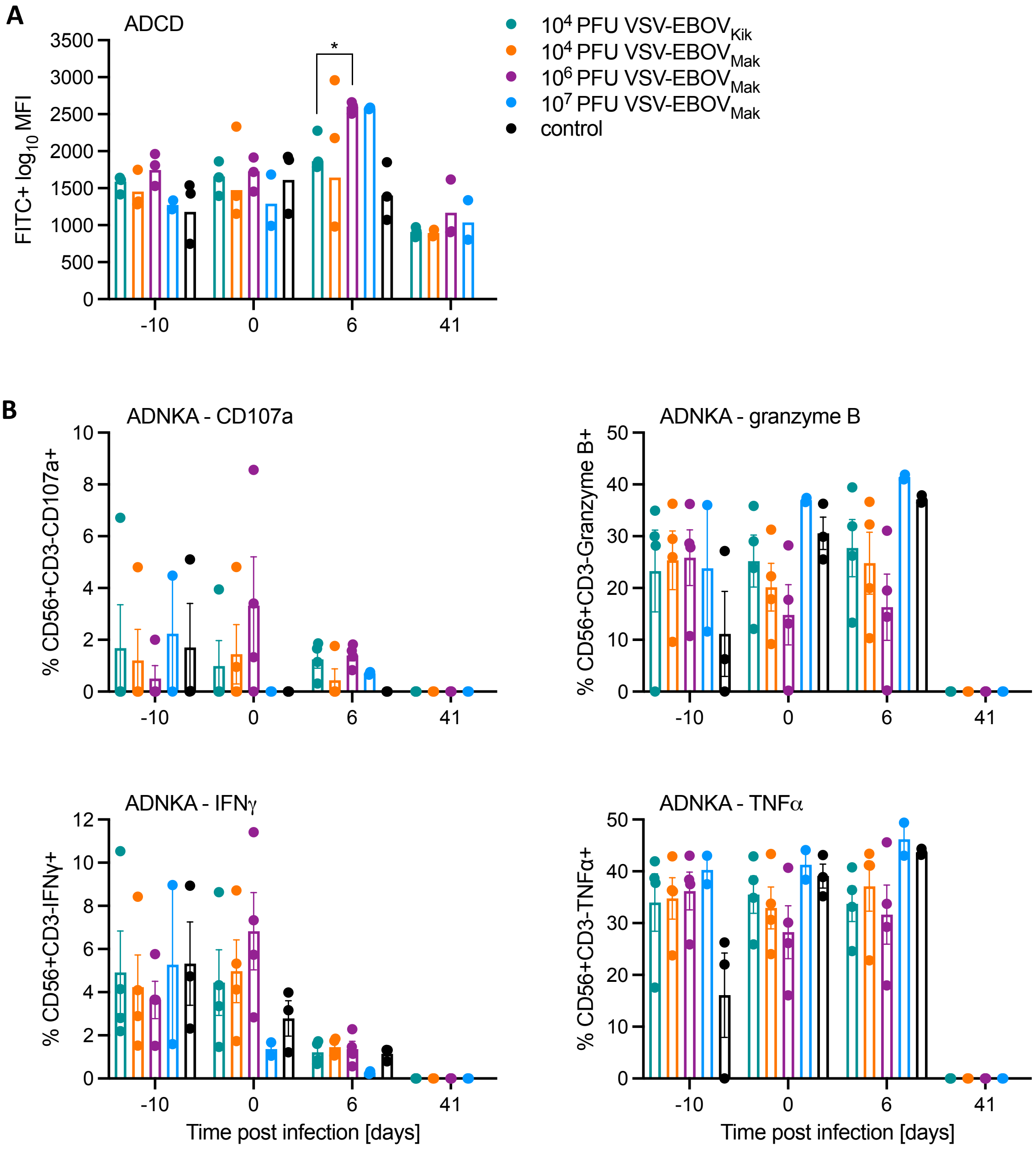

A
ADCD
FITC+ log_{10} MFI
3500
3000
2500
2000
1500
1000
500
0
*
-10
0
6
41
10^4 PFU VSV-EBOV$_{Kik}$
10^4 PFU VSV-EBOV$_{Mak}$
10^6 PFU VSV-EBOV$_{Mak}$
10^7 PFU VSV-EBOV$_{Mak}$
control
B
ADNKA - CD107a
% CD56+CD3-CD107a+
ADNKA - granzyme B
% CD56+CD3-Granzyme B+
ADNKA - IFNγ
% CD56+CD3-IFNγ+
ADNKA - TNFα
% CD56+CD3-TNFα+
Time post infection [days]
Time post infection [days]

Figure S4

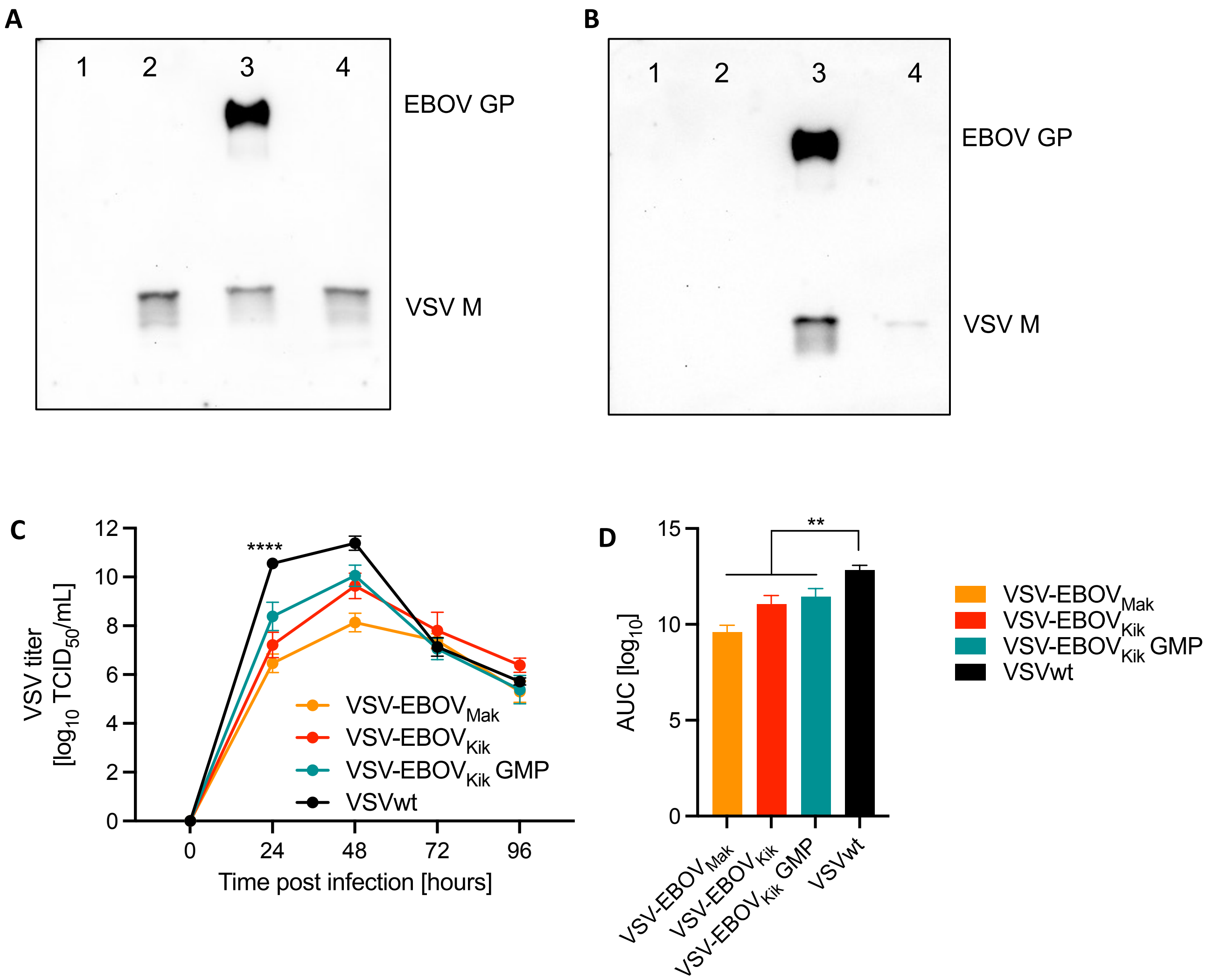
A
1
2
3
4
EBOV GP
VSV M
B
1
2
3
4
EBOV GP
VSV M
C
****
VSV titer
[log10 TCID50/mL]
0
2
4
6
8
10
12
0
24
48
72
96
Time post infection [hours]
VSV-EBOVMak
VSV-EBOVKik
VSV-EBOVKik GMP
VSVwt
D
**
AUC [log10]
0
5
10
15
VSV-EBOVMak
VSV-EBOVKik
VSV-EBOVKik GMP
VSVwt
VSV-EBOVMak
VSV-EBOVKik
VSV-EBOVKik GMP
VSVwt
